# Appreciating the complexity of frailty and user context in digital health intervention design: A qualitative study with personas

**DOI:** 10.1371/journal.pone.0343371

**Published:** 2026-04-06

**Authors:** Jenny Corser, Laura Dennison, Lizzie Coles-Kemp, Helen Dawes, Maedeh Mansoubi, Glen Cooper, Andrew Weightman, Athia Haron, Felicity L. Bishop, Sarah E. Lamb, Alison Pighills, Annie Clewlow, Marcia Hudson, Emily Lam, Philip Elphick, Robert Ingle, Tressa Davey, Katherine Bradbury

**Affiliations:** 1 Centre for Clinical and Community Applications of Health Psychology, University of Southampton, Southampton, United Kingdom; 2 Information Security Group at Royal Holloway, University of London, London, United Kingdom; 3 Medical School, University of Exeter, Exeter, United Kingdom; 4 University of Manchester, Manchester, United Kingdom; 5 Mackay Hospital and Health Service, Mackay, Queensland, Australia; 6 Communicare in Southampton, Southampton, United Kingdom; 7 Patient and Public Engagement Group, Southampton, United Kingdom; Instituto Nacional de Geriatria, MEXICO

## Abstract

Frailty is a complex and dynamic process that is becoming increasingly common as the population ages. Early intervention has been shown to reverse or slow progression, but requires support from increasingly limited healthcare resources. Digital health interventions could potentially assist, but first it is vital to understand older adults’ experiences and perspectives. This study describes feedback from older adults with symptoms of pre or early frailty on the design concept of a digital device intended to function as an adjunct to physiotherapy and occupational therapy by increasing frequency and quality of safe movement at home. Objectives were to better understand experiences of frailty, and to identify potential influencing factors on adoption and adherence. In-person audio-recorded qualitative interviews were conducted with 17 older adults and 5 carers recruited from community settings. Reflexive thematic analysis generated two themes: ‘autonomy and prompts: importance of personalisation’; and ‘digital interfaces and accountability: importance of usability and purpose’. In addition, three personas were generated from the data, each describing different needs and preferences for such a device. Situating the results of analysis in the context of current literature, a set of Guiding Principles was created to support future design development in this area. These highlight important factors for consideration when designing for this population, and provide key design feature suggestions and implementation strategies to address these factors including: ensuring the device has clear purpose; is adaptable to the diversity of needs within this clinical population; any form of feedback is safe, relevant, useful and rewarding to multiple users; and technical support is accessible and ongoing. It is recommended that the target population, and clinical purpose be more clearly defined within the healthcare context to encourage personal rehabilitation through enhancing the therapeutic alliance rather than focussing on individual behaviour change of diverse older adults.

## Introduction

Frailty is a common and important health issue for older adults with an overall UK prevalence estimated to be 8.1% among adults over 50 years old, with substantial variation by geographic region, with the lowest regional prevalence at 4.0% and the highest at 15.7% [1]. With an ageing population and increasing number of people living with chronic disease, the prevalence and associated health costs of frailty are rising [2]. There is no gold standard definition, but it is generally understood as an age-related state of reduced physical and social robustness, and consequent increased vulnerability to adverse health outcomes [3–7]. It is commonly measured using either the Fried frailty phenotype focussed more on symptoms such as loss of strength, inactivity and fatgiue [4] or the Frailty Index with a greater emphasis on multimorbidity [8]. More recently, there is a call to broaden these definitions of frailty according to the international classification of functioning, disability and health framework to include physical, mental, personal, environmental and social factors [9].

Risk factors for frailty are multifactorial including sociodemographic and psychosocial factors, and prevalence is consistently higher for women than for men [10]. Few longitudinal studies examine the progression of frailty [10]. Frailty can be conceptualised as a process of decline, preceded by prefrailty: a clinically silent process, or latent phase, where people have one or two of the symptoms that later classify them as frail, predisposing individuals to frailty [3]. Pre-frailty presents as an intermediate state between robustness and frailty, where risk of frailty is higher [11], offering optimal timing for preventative rehabilitation [12]. As such, we must understand frailty as a dynamic multifaceted process requiring ongoing, holistic person-centred evaluation [13–15].

Rehabilitation for frailty and pre-frailty includes supporting people to increase their physical activity levels, particularly through resistance-based strength training [16]; critically, movement interventions that are wholly self-led are less effective than supervised interventions [17]. Older adults living with frailty have significant barriers to increasing physical activity, including loss of confidence (particularly with fear of falling [18]), pain, fatigue, low mood and lack of awareness of appropriate and beneficial exercises [19]. The literature emphasises that older adults’ attitudes towards exercise are generally positive, and that having guidance from a professional (e.g., physiotherapist) greatly increases self-efficacy and engagement [19,20]. Occupational therapy assessments of the person’s home environment can help reduce the risk of falls and facilitate improved confidence and functionality within the home and home-based activities [21,22]. Unfortunately, access to these services is often limited. According to the British Geriatrics Society, 81% of services for older adults routinely face workforce shortages on a weekly basis and 51% on a daily basis [23]. Digital technologies may help improve access to physiotherapy and occupational therapy support.

Digital health interventions offer potential to monitor personal health changes while supporting the implementation of rehabilitation services to reach large populations without using as much healthcare practitioner resource, which is often limited. The ability to collect naturalistic, longitudinal data to assess abilities over time is especially relevant to the dynamic nature of frailty [15]. Digital devices can also be helpful to improve social connectivity, and promote physical exercise, while unobtrusive body worn sensors around the waist, for example, can predict frailty, slowness, performance and physical activity [24]. Current devices focus on individual aspects of frailty, but as yet no studies combine multiple measures, or explore how people are moving (e.g., gait asymmetry) in the complexity of naturalistic conditions [25]. A scoping review of current technologies targeted towards frailty highlights the tension between standardising frailty measures and requiring personalised interventions to address individual variation, emphasising the need for devices to be co-designed and methodologically tested [26], and there is a call for qualitative research to explore lived experiences of frailty to better inform intervention development and delivery [27].

The current study explores older adults’ and their carers’ perspectives on a specific design concept: a novel digital intervention intended to provide some of the support that occupational and physiotherapists would ideally give to help rehabilitate people with pre-frailty. The proposed technology integrates multiple wearable sensors, to be worn daily to collect naturalistic information on the user’s gait, stability and physiological response to movement, with a user interface that prompts personalised exercises designed to improve measures of frailty by regaining or maintaining strength and stability. The second aspect of the intervention design includes 3D modelling of the home environment using light detection and ranging technology (LiDAR) to facilitate a home assessment by an occupational therapist without the need for a home visit, and elements of social connectivity, and the design and development is being led by feedback from older adults. In this early phase of the development, potential users (involved in the study via formal participant, and patient and public inclusion and engagement (PPIE) groups) acted as critical friends to provide in-depth feedback on the initial design proposal to allow intervention planning. This paper presents an in-depth qualitative study which aimed to collect feedback from older adults who identify as starting to have difficulties moving (pre or early frail) on the proposed device as part of an overarching co-design methodology, the Person-Based Approach [28]. The objectives were to better understand experiences of frailty both thematically and through the creation of evidence-based user personas (archetypes of potential users of the proposed technology that are used to guide technology development), to identify user preferences, as well as influencing factors to adoption of and adherence to such a device.

## Methods

### Study design

This study describes the intervention planning phase of the Person-Based Approach [28]. The Person-Based Approach combines user centred design and behavioural science methodologies to generate in-depth qualitative data to better understand and accommodate the needs and preferences of target users of an intervention. This study involved qualitative data collection via semi-structured interviews conducted in person, in the participants’ homes, alongside regular PPIE meetings with a group of seven members of the public who either identified as prefrail, or cared for someone who was frail, or worked in the community as a volunteer providing support to older adults. A PPIE group acts as a committee of people with lived/living experience of the subject being studied who can provide vital insight throughout all research processes to minimise stigma, and maximise credibility and relevance.

The data gathered during the interviews were thematically analysed to describe the researchers’ interpretation of findings. In addition, user archetypes were created grounded in a grouping of the different needs, attitudes and capabilities identified from the data. These archetypes are a methodogy used in the field of human-computer interaction and are termed “personas” [29]. These were used to communicate variations within a group of potential technology users to further guide user-centred technology design.

Drawing on the in-depth understanding of the target user group from thematic analysis and persona generation in the context of current literature, Guiding Principles were generated using the Person-Based Approach [28]. These are design principles focussed on making an intervention engaging, persuasive, meaningful and enjoyable in order to optimise user uptake, engagement and sustained use required for improved clinical outcomes. The Guiding Principles therefore describe implications of the primary data and are presented in the Discussion section to communicate key design features and implementation strategies for this device and others like it.

Ethical approval for this study was obtained from the University of Southampton (ERGO# 78959.A1).

### Participants

Recruitment was focussed in and around a large urban/coastal area in the south of England and was open from 9^th^ May 2023 and closed 15^th^ December 2024 due to study timelines. Engagement with local community partners (charities focussing on older adult wellbeing through befriending or provision of activities) was essential to recruitment. A recruitment flyer was co-designed with a local good neighbour organisation, Communicare, for postal distribution via their monthly newsletter. The flyer invited people aged 65 or older who were finding walking harder, more tiring, or felt like they were losing strength, to take part in an interview to discuss the project and their thoughts about the proposed technology. The invitation noted that all participants would receive a £20 gift voucher in gratitude for their time, and that if interested they could call or email named researchers for more information about the study, and to provide contact details for participation.

Individuals interested in taking part contacted the research team directly and were screened for eligibility by telephone. Eligible participants were over 65 and scored 1 or 2 (prefrail) on the simple frailty questionnaire [30]. Participants who scored less than 1 (not frail) or more than 3 (very frail) were excluded. This questionnaire is less detailed than longer form screening tools, and requires further diagnostic validation, but incorporates elements of the frailty phenotype while also accounting for multimorbidity and has been shown to be a time and cost-effective screening instrument for frailty [31]. It does not measure cognitive frailty, and we did not exclude participants who self-reported cognitive impairment. All participants had capacity to consent to the study which was assessed during screening and interviews as per the UK Mental Capacity Act (2005) [32]. Participants were also selected purposively to include a range of age, gender, ethnicity, relative deprivation (based on Index of Multiple Deprivation score [33] from address) and health literacy (using a brief questionnaire [34]). Based on previous projects, a sample size of 20 older adults and family caregivers was estimated to provide sufficient information power [35] for the study. Eligible participants were invited to attend an interview in person in their homes (or elsewhere if preferred).

Recruitment stopped once the target of 20 older adults was reached, unfortunately, 3 were subsequently lost to follow up, where contact was made in this situation it was found to be due to hospitalisation. During the recruitment period, 2 were deemed ineligible due to permanent disability rather than age related frailty, 6 scored as robust when screened, and 1 declined to take part after being provided further information due to their dementia and feeling it was inappropriate for them. A total of 17 older adults (7 male and 10 female) and 5 family caregivers (3 were spouses and 2 were children of the older adults) were recruited. The average age of older adults was 79.7 (SD 5.5) years. Eighty-two precent of older adults identified as white ethnicity, and 17.6% identified as black ethnicity; of these, 64.7% lived alone, and 58.8% scored as frail. Participant characteristics are summarised in Table 1.

**Table 1:** Participant characteristics.

Characteristic	N(%)/Mean ± SD
Participant type:	
Older adult	17
Carer	5
Carer relationship:	
Spouse	3
Child	2
Older adult gender:	
Male	7 (41.2)
Female	10 (58.8)
Carer gender	
Male	1 (20)
Female	4 (80)
Older adult age, years	79.7 ± 5.5
Older adult ethnicity:|	
White British	14 (82.4)
Black/African/Caribbean	3 (17.6)
Carer ethnicity:	
White British	4 (80)
Black/African/Caribbean	1 (20)
Index of Multiple Deprivation score	4.9 ± 2.9
Older adult housing:	
Lives alone	10 (58.8)
With at least one other family member	6 (35.3)
Accommodation with warden on premices	1 (5.9)
Older adult education:	
High school	11 (64.7)
College	3 (17.7)
University	3 (17.7)
Frail Scale Score^1^:	
O (robust)	0
1 (prefrail)	4 (23.5)
2 (prefrail)	3 (17.6)
3 (frail)	10 (58.8)
4 (frail)	0
5 (frail)	0
Health literacy score^2^:	
12	3 (17.6)
13	1 (5.9)
14	9 (52.9)
15	3 (17.6)

^1^Measured using the simple frailty questionnaire [30].

^2^Measured using a brief questionnaire [34] with a maximum score of 15 indicating all participants had adequate health literacy.

### Data collection

In-person interviews were conducted in the participant homes by LD (an experienced female qualitative researcher). Each participant was interviewed once. Interviews were conducted one-to-one except for older adult and caregiver dyads who were interviewed together.

Prior to audio-recording, the researcher reviewed the purpose of the study and provided a participant information form. The participants were given the opportunity to ask questions and then asked to complete the written consent form.

A semi-structured interview guide with main questions and prompts was used to guide the research (see Supporting Information 1). Interviews began by asking about the participants’ general routine, any challenges moving around, and what they enjoyed doing. Participants were asked about any mobility aids they might use and if they had any prescribed exercises they do on a regular basis. The researcher then provided a standardized lay summary of the concept of the technology design for feedback, highlighting that the concept is still in very early exploratory stages, and there was no prototype to show them, but currently available health technologies were used as reference such as smart watches (see Appendix 1). Participants were asked about their first impressions, whether they could imagine themselves using the device, how they felt about different elements of the design, and if they had any concerns or preferences. Interviews lasted an average of 79.4 minutes (SD 18.2) and were audio recorded and transcribed verbatim. In addition to interview data, the interviewer made field notes and reflections about their conversations with the participants and observations in the home.

### Data analysis

Data were collected in parallel with ongoing device development and were initially coded for technical feedback for the engineering team, to directly inform their design. A brief summary of these findings is presented in Supporting information 2. Once all data had been collected, JC (an experienced qualitative researcher, behavioural scientist, and lead author) analysed the data as a corpus using reflective thematic analysis [36]. This analytical approach is appropriate for inductive exploration of qualitative data from a constructivist viewpoint acknowledging the importance of context in understanding, including the implicit role of the research team in the analytic process and can be used flexibly [37].

Data analysis began with reading over the transcripts and field notes to gain familiarisation with the data. Each transcript was coded inductively using NVivo software [38] and reflective notes taken throughout the process. This was an iterative analysis leading to the generation of themes to draw out key information to better understand contextual issues and user preferences important to informing the device design. These themes were discussed and refined with the author’s line manager KB (an experienced qualitative researcher and health psychologist) and presented to the wider research team (including engineers, and healthcare professionals) and PPIE group for discussion and further iteration. The final themes are described in the Results with anonymised illustrative quotations and reflect a collaborative interpretation of the data collected *Personas.*

In addition to generating overarching themes to illustrate participants’ perceptions of the technology, personas were created to highlight three unique participant groups identified in the data. The persona creation was a separate, additional, analysis aiming to illustrate the impact of different context within this patient group (which clearly illustrates how different barriers/enablers apply in different situations). Whilst this analysis was separate, the same reviewer conducted this, so both analyses would have informed each other. These personas illustrate key differences within the potential technology user base and demonstrate the impact of contextual factors on the understanding and acceptance of, and potential engagement with the device being developed. Personas are a standard technique in human computer interaction (HCI) [29] with a practical rather than theoretical approach. These personas were identified through immersion with the data (including transcripts, field notes and researcher reflections) using constant comparison of key contextual differences and similarities between participants. No prior inclusion/exclusion criteria were used, rather this was an inductive process of identifying patterns in the participant characteristics and how they related to the proposed device. Key elements of the personas included lifestyle, attitudes to their health and wellbeing, degree of comfort and confidence in using digital technologies, and personal circumstance (home living arrangements). The approach looks at each participant account individually in terms of the chosen criteria, and then grouping similar patterns together to form an archetype describing a group of similar shared characteristics. While the themes discuss wider issues relating to the technology for the group as a whole, the descriptions of the personas identify key barriers and opportunities in different contexts within the sample. Personas were developed by the main author JC, with input from KB and LC-K (an expert in HCI), and the PPIE group as a means of creating accessible feedback for the technology developers and intervention designers. Personas are also a means of challenging design assumptions. Whilst the technique of using personas has been critiqued for being reductionist and having the potential to re-enforce biases in attitudes towards a potential technology user group [39], personas were used in this study because they created a means of illustrating the voices of frailer, older people in a format that technology and intervention designers found accessible and compelling.

In order to ensure quality of reporting, the consolidated criteria for reporting qualitative research (COREQ) was followed [40]. A copy of the checklist, including reflexivity statement, is included in Supporting information 3.

## Findings

Participants spoke about their general attitudes towards improving their mobility, and experiences with technology in ways that implicated potential areas for consideration for the development of a digital intervention involving wearable sensors with an interface to prompt strength-based exercises and the use of digital mapping technology for occupational adaptations of the home. Analysis of the data generated two themes: ‘autonomy and prompts: importance of personalisation’; and ‘digital interfaces and accountability: importance of usability and purpose’. These themes are illustrated below, followed by a summary of three personas identified in the sample.

### Autonomy and prompts: Importance of personalisation

Participants prized their independence, and their motivation to increase their activity levels centred around this concept. Their independence was described in terms of both physical mobility and cognitive function, and any sense of losing independence generated emotions including grief, frustration, anger, and sadness. Retaining, or rebuilding independence and autonomy was a motivation to consider this kind of supportive intervention as well as a condition of using it (i.e., the intervention had to respect their independence in the capacity of its role in supporting it). It was important for the participants to be able to personalise the device in various ways, particularly for it to be programmed to fit in with their personal lifestyles, without being intrusive, in a way they felt autonomous and in control.

Most participants were amenable to the idea of wearing sensors, as long as they were not too physically cumbersome, uncomfortable, or fiddly to put on or wear for long periods. The idea of being prompted by a device to exercise, however, generated more concerns of invasiveness or intrusion.

“I’m very, very comfortable with my life. I don’t really want anything to interfere with that. It’s a nuisance. So I would probably give it a go, but may take it off!” (P12, 80, frailty score 3, lives alone)

While some participants enjoyed participating in public or private formal exercise programmes, most of the activity they described was functional (e.g., walking up and down the stairs, cooking for themselves, bathing, cleaning, shopping). As such, the idea of being prompted to do prescribed exercises was interpreted in different ways. Participant 11, a woman living alone in a three-storey home and walking up and down the stairs every day, stated that despite her active lifestyle, she felt that she ought to be doing formal exercises, “I don’t do enough actual exercise like they keep saying to. That’s what I should do.” (P11, female, 86, frailty score 3, lives alone). Participant 6, however, who had recently moved from a two-story house into a bungalow, felt that she would prefer that the device could recognise that it would not be appropriate to prompt her to exercise on her more active days when she would be too tired.

“Sometimes, when you go shopping, you’re exhausted in the afternoon. You don’t want to do a load of exercises. Perhaps if it had a device that would trigger that you wouldn’t have to do a load of exercises if you’d done 1000 steps in the morning because it’s too much” (P6, female, 68, frailty score 3, lives with and cares for spouse)

Participants also had specific preferences in terms of how prompts for exercise would fit in with their daily routines and energy levels. Each person had clear times of the day when they were more productive, and these were highly individual. Some participants were able to list all their daily activities as a regular routine, while others were less habitual. Likewise, factors such as meals, social interactions, mood or medication use could have an impact, and each participant felt that the device would have to be attuned to their personal preferences and energy cycles.

“I have to do it before breakfast because I’m a bit lazy during the day. If I did it after breakfast, I’d never get to do it. So, I have my cup of tea in the morning, and because I take the drugs at night I’m a bit woozy in the morning. Takes me about an hour to come round. So, I watch a bit of television, then I go and get washed, and then I do my exercises, then I’m ready for my breakfast, which is usually quite late but there you go.” (P8, female, 73, frailty score 3, lives alone)

Some participants felt that prompts would be beneficial, and would help nudge them to action. They felt that prompts would serve as reminders and encouragement to move, especially if there was an element of fun or reward. The idea of how the prompts would work were interpreted in different ways. One participant associated prompts with how he felt about music and how it organically prompted him to dance, something he greatly enjoyed.

“Well, you need to be prompted, don’t you, really? You need something to prompt you, whether it’s the sound of the music or because, for instance, if it is anything I’m watching on the TV and the music sounds good, I’ll get up and start moving to it. It’s ways of prompting you to get up and do something.” (P16, male, 82, frailty score 1, lives with child and grandchild)

Several participants associated prompts with interfering with their autonomy to decide when to move for themselves. Their reaction to imagining a device prompting them to stand up was sometimes quite dismissive and assertive, “No, I’ll tell myself when I want to stand up!” (P2, female, 85, frailty score 1, lives alone). It was as if the suggestion to have a device prompt them into action was insulting to their independence, “it might help somebody who’s not quite with it” (P1, female, 8-, frailty score 1, lives alone). One suggestion to mitigate this feeling was to compose the messaging in a more gentle manner, as a suggestion rather than a demand.

“I resent people telling me to do something. Asking me is a different matter. ‘Do you think you could stand up, [name of participant]?’ Possibly, yes, I can give it a go. ‘Do it now,’ - piss off!” (P12, 80, frailty score 3, lives alone)

The option to provide input into the system in order to mute it temporarily or postpone activities was proposed by some participants. Fatigue, mood, and pain were key factors that were suggested as important inputs that people would like to provide. Family caregivers were already navigating the balance of respecting the older adult’s autonomy while providing support. These participants felt that receiving feedback from the older adult about their daily movements (or lack of movement) would be helpful as they would not have to keep checking in directly. One caregiving participant suggested a traffic light system that could serve as a means for the older adult to have some control over the prompts, but also to provide objective feedback to the caregiver as reassurance or indication to follow up.

“if you could incorporate something that just said, very simply, red: I’m feeling rubbish. I can’t do very many exercise today, I’m just pressing red. Then amber: not so good. Green: I’m ready to go. It’s a very simple way of getting that message over quickly, and if you build that up over a series of days, it then gives you a rough idea of how a person might be ticking.” (P17, family caregiver)

### Digital interfaces and accountability: Importance of usability and purpose

The participants reported varying levels of engagement and confidence with technology from only using their television, to navigating the internet and using various apps on multiple personal devices. Their relationship with any device was directly related to their sense of self-efficacy or control, and hence their ability to use it to their advantage rather than feel overwhelmed by it. This theme, therefore, follows from the first that highlights a need for a digital intervention to respect rather than threaten the user’s independence and sense of autonomy, to a focus on the user perception of the device as useable with clear purpose.

Even the participants who described themselves as insecure about technology had some sort of device that they used on a regular basis, and they were comfortable with these tasks because of their familiarity with the specific interface and ability to achieve their related goal (self-efficacy). Whether that was finding the right channel, completing an online shop, or checking the weather, they seemed to feel in control of these tasks rather than vice versa. When applications became too intrusive (too many notifications), or changed too frequently (too many updates), participants reported frustration and disengagement. Likewise, participants tended to feel anxious about and avoid situations where they struggled to navigate the system or go back if they pressed a button in error.

“No, I’m just a bit worried about anything that has any tech in it! I press a button, and that’s why I don’t do banking online, because I press a button, and everything disappears sometimes, so I think, where’s it all gone?” (P6, female, 68, frailty score 3, lives with and cares for spouse)

“Oh, I don’t touch it. I don’t touch it because I just worried if I touch it, I might do something wrong.” (P14, female, 82, frailty score 2, lives with spouse)

Even the most insecure participants were open to learning new systems, but were not always used to having adequate tech support available to spend the time to explain the device. Likewise, some participants noticed a decline in capabilities, either due to technology evolving, or due to cognitive changes, so ongoing support to maintain their longer-term relationship with technology would be critical.

“it’s just got so complicated. I’ve forgotten it. It’s just so soul-destroying, in a way. That’s why I’m very aware I don’t try anymore… The things that I used all the time, did Tesco orders, somehow have gone. I guess because I can’t deal with the tech bit. It’s very frustrating.” (P5, female, 76, frailty score 3, lives alone)

Purpose was also key to good engagement with technology. Every device interaction participants described was to achieve a specific goal (shopping, communicating with others, playing music, taking photographs). Where there was familiarity and self-efficacy, these functions were highly valued, “I’d be lost without my laptop” (P8, female, 73, frailty score 3, lives alone). Some participants had experience with health devices such as health monitors and pacemakers, trackers, and panic alarms. The purpose of these devices was clear, and where there was positive experience or feedback as a result of using them, their value increased.

“that was how I got this one [panic alarm]. I find myself now, because I’ve had two or three falls. Nothing too serious. Each time now, if I’m - sometimes if I’m going down in a lift or whatever I’m doing, and I automatically go like this [touches her alarm] to make sure I’m wearing it. So I’m aware now that it is very, very useful.” (P3, female, 86, frailty score 3, lives alone)

The aspect of the technology that seemed to be the most confusing or elusive to the participants was the use of Lidar to model the home. This technology is often used by real estate agents from smartphones using an application and this example was recognised by some participants, though none could comment about this in much depth. We discussed that the application could possibly be downloaded to a smartphone belonging to them or a family member for home assessment without the need for a visit from a healthcare practitioner, but we did not discuss how the technology operates. Participants were often familiar with home adaptations that result from home assessments, though sometimes the benefits of home adaptations were only apparent after they had been installed “I wondered why they put those rails in, but now I’ve got older, I can see why – they’re brilliant. I wouldn’t have a bath without those rails there now, no way” (P11, female, 86, frailty score 3, lives alone). This retrospective appreciation again suggests that the mechanisms of the assessment phase were eventually accepted as part of their care pathway, even if the process appeared unclear at the time.

Participants interpreted the purpose of the study technology in different ways. As well as wanting it to be personalised to them (see first theme), they needed to understand what they were meant to achieve out of it, “it’s got to be very specific to Dad and it’s got to be intuitive enough to know what are we going to get out of this?” (P13b, family caregiver). Some participants saw it as a prescribed device to help healthcare professionals improve their diagnosis and treatment, indicating a power imbalance and adherence in response to the perceived authority of professional advice “doing what you’re told” (P1). In this way the benefit of the device to themselves was indirect, supporting the healthcare professionals first, to improve their care second.

“I don’t think you get too many people volunteering [if told to wear it all the time], but then, if you’re not volunteering for it, if it is part of your health programme, or what they want you to do, you would hopefully, say, oh, well, all right, if it’s going to help me, I’ll do it, yes.” (P1, female, 80, frailty score 1, lives alone)

Likewise, another the benefit was seen as reducing the burden on health services in general. For example, the few participants who were enthusiastic about the 3D modelling of the home, were motivated by their curiosity of advancing technologies and the opportunity to take a task off the hands of overburdened health workers.

“it save the person coming in and doing that, doesn’t it?... Then if we got stuck, then we’d say, well we’ve tried it and it didn’t work. Again, that side of it would help the professionals if we could do it, it’s time saved and effort, isn’t it? So we’d have a go, quite happily, wouldn’t we?” (P17a, family caregiver).

Participants who attended social fitness groups, or had experience of physiotherapy treatment related the device to these experiences. Although personal home exercises lacked the sense of belonging and social support gained from these groups, the importance of being active was recognised. Adherence to exercises in these situations was enhanced by a sense of accountability to the group facilitator, and similarly, with the device idea, there was a feeling that there might be someone monitoring their activity who would know if they did not follow the instructions.

“you do it because you know you ought to do it, and they’re going to ask. I suppose if your watch was taking your data, you’d also do it because it was taking your data!” (P6, female, 68, frailty score 3, lives with and cares for spouse)

The idea that there was some sort of human interpretation or monitoring of the device was an essential element of acceptability. Even though participants had not experienced continuity of care or regular in-person interactions within current healthcare teams, the idea of this technology being automated did not always sit well, “So I’d end up being a robot?” (P13, male, 88, frailty score 3, lives alone) and there was a strong preference not to become overly reliant on technology at the expense of valued human interaction. That said, the possibility of constant monitoring was seen to be a realistic improvement and provide the continuity of care that was currently lacking – as long as there was still someone on the other end to interpret and respond to the information.

“Well, I wouldn’t say it’s a bad idea, because if it’s something you’re going to wear to pick up information to let you know, or let whoever is doing it know what is best for that person, then I wouldn’t say - that’s not a bad idea, that’s a very good idea.” (P14, female, 82, frailty score 2, lives with spouse)

There was a recognition that digital devices could reduce the burden on the stretched healthcare system, and the intention of the device was understood. Likewise, some assumed that it might be the family caregiver who managed the information, and appreciated their need for support. Participants who were caregivers valued the idea of anything that might reduce the burden of their caregiving and felt reassured about an intervention designed to support safe exercise at home, especially if it did not create another task for them to have to monitor or troubleshoot. Again, the expectation was that the ultimate purpose of the device was as a data collection or communication tool for healthcare professionals.

“The emphasis would be on me again, but I quite like the idea of having that layer between me and the health professional. You do, because the default is GP, because I don’t know, I’m not medical.” (P13b, family caregiver)

### Personas

When designing digital interventions for clinical populations, user context is a vital consideration alongside clinical targets [41]. Within our sample of 17 older adults self-identifying as finding it harder to move around, and scoring 1–3 out of 5 on the simple frailty questionnaire [30] we found three distinct technology user archetypes, or personas, each operating within different contexts of technology use with unique needs in relation to the proposed technology.

#### Persona 1: Julia.

**Fig 1:**
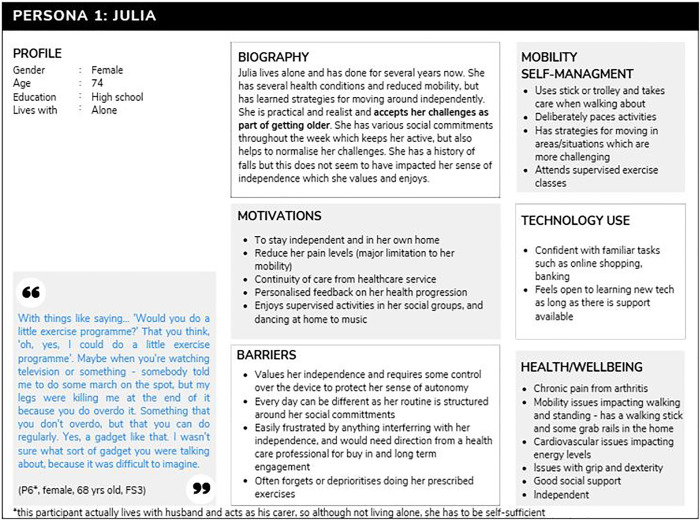
Persona 1, Julia.

This persona was the most common in our sample (n = 9). Julia is independent and has a loose routine revolving around her social commitments, which remains flexible. She accepts her identity as an older person, and puts any health concerns down to the natural process of getting older. She gets herself around using public transport or lifts from friends. Although she may have a history of falls, this does not appear to have impacted her confidence to get around independently. Julia is enthusiastic about the idea of the technology to help healthcare practitioners provide better, more consistent care. She imagines it would be a temporary device, worn periodically to help her healthcare professionals with their diagnosis and treatment plans, in the same way that she has experienced using blood pressure monitors from her doctor in the past. While this use of the device is purposeful and acceptable to her, the idea of wearing a device long term for consistent monitoring outside of an active treatment plan was not, and the concept of being prompted to move by a machine is seen as an insult to her independence. Fig 1 outlines Persona 1 (Julia).

#### Persona 2: Patrick.

**Fig 2:**
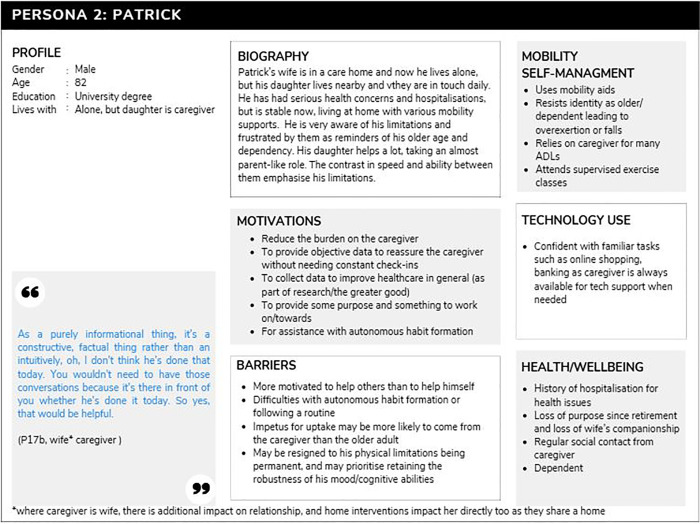
Persona 2, Patrick.

The persona of Patrick (n = 4) is dependent on his overwhelmed, and possibly quite dominating family caregiver. The dynamic of carer and cared-for has put a strain on their relationship, and the contrasts of speed and ability make the limitations of the cared-for older adult more obvious. This adds to feelings of frustration and inadequacy on the part of Patrick, and anxiety hypervigilance for his caregiver. Patrick has a history of serious health scares and hospitalisations. He and his caregiver are anxious and worried about further decline in health, and where the caregiver is also an older adult, there is concern that they will not be able to cope. Patrick’s caregiver often monitors and encourages health behaviours so that he is not motivated to form habits for himself. He resists his identity as old and may overestimate his abilities by over exerting himself while trying to do things he used to be able to. This makes his caregiver more anxious and vigilant. There is a strong sense in this context that Patrick’s declining health is putting a burden on the household and home adaptations designed to help him may act as a further reminder of this. Patrick and his caregiver are interested in the technology to help relieve the caregiver or act as a reassurance of safety when they are not around. This persona tends to be more confident with technology in general because of caregiver influence to introduce it and be available for tech support when needed. Fig 2 outlines Persona 2 (Patrick).

#### Persona 3: Christine.

**Fig 3:**
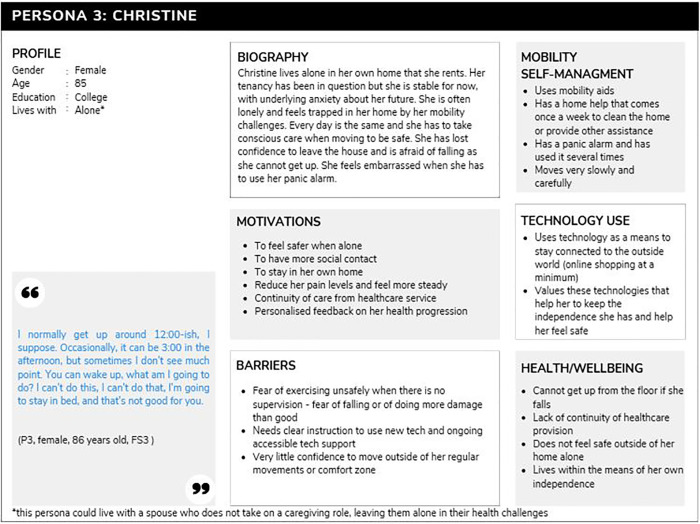
Persona 3, Christine.

This persona (n = 4) tended to score higher on the frailty scale. Christine is lonely, she feels she has to accept her situation and remain optimistic, taking each day as it comes. She has limited mobility, and is reliant on others to do anything, and where she has no one, she is isolated, almost trapped, in her home. She is highly routined, every day is the same. She is quite scared of her declining health and has lost confidence in her mobility, often describing a fear of falling and not being able to get up. Christine uses digital devices to connect with outside world (e.g., online shopping, skype) but needs accessible tech support provision to maintain this. She would be interested in tech that might connect her with others as her strongest need is a sense of belonging and safety. This persona often uses a panic alarm for these purposes and was interested in a device that could reliably guide her to move safely and reassure her, as well as incorporating some sort of group activity or buddy system to connect with others outside of her home. Fig 3 presents Persona 3 (Christine).

## Discussion

This paper explores feedback from older adults and their family caregivers on the design concept of a novel digital system designed to support prefrail-frail older adults regain strength and stability. Rigorous analysis of transcripts, field notes, and diverse PPI involvement generated themes describing complexities vital for consideration in digital device design, and three personas illustrating diverse needs within this population.

Within the themes, several tensions were noted at different levels of perception when thinking about the device. Personally, the device had to support independence while remaining physically and cognitively unintrusive or insulting a sense of autonomous personhood; in terms of relationship with the technology itself, there had to be a sense of control over the technology and clear purpose for it to be seen as a tool to help them achieve their needs; and more widely, participant feedback appeared to be influenced heavily by interpersonal factors such as presence of, or relationship with, a family caregiver. This latter concept raised unexpected motivational factors such as needing a sense of purpose, wanting to oblige the researcher or healthcare professional’s requests, or relieving caregiver burden. These factors describe contextual, and ethical considerations that must be considered and prioritised when designing assistive technologies for older adults to facilitate behaviour change, yet are often overlooked in research [42].

The focus on user context allowed for a wider scope of discourse about the device concept and importantly brought the voices of our participants into direct conversation with the technology and intervention designers. These voices were often gently critical and challenged some of the core assumptions that had shaped the intial intervention prototypes. Crucially, the importance of personal support alongside the technology was emphasised repeatedly and has salient implications for critical reflection on the device design purpose. Participants expected there to be human support in several areas of the device including 1) the introduction of the device and encouragement to maintain adherence by a healthcare practitioner; 2) the provision of data reports to be fed back to a caregiver for periodic monitoring and reassurance; and 3) the ongoing availability of technical training and support to use the device. These preferences need to be considered by technology developers and policy makers motivated to create technological solutions aimed to reduce the burden on the healthcare system. Additional costs of support staff and training associated with a digital health system in this population could mean that the anticipated relief of burden to the health system actually becomes a transfer of burden to the technology distributer or user that needs to be acknowledged and accounted for [43]. Family caregivers and health practitioners play important roles in influencing older adults to adopt and adhere to health technologies and should collaborate in the decision-making processes during design, but also for implementation of the device. A recent scoping review of assistive technologies for older adults echoed some of the reciprocal motivations noted by participants in this study, namely: to improve their independence, reduce caregiver burden, and demonstrate commitment to rehabilitation [43]. This highlights the important roles of relationship, altruism, and accountability, that directly impact user uptake and engagement with a device, and stress the need to involve multiple stakeholders at all stages of the design and implementation.

There is currently a scarcity of clear design principles relating to the development of technology for home-based rehabilitation in older adults [44]. The Guiding Principles developed here (see Table 2) are derived from this contextual and thematic exploration of a diverse sample of frail older adults and focus on areas relating to design optimisation and successful implementation. Key principles include ensuring the device has clear purpose and is adaptable to the diversity of needs within this clinical population; any form of feedback is safe, relevant, useful and rewarding to multiple users; and ongoing technical support is provided to mitigate changes in self-efficacy with device interaction. These align with recent design principles developed by Schlieter et al. (2024) for home-based “virtual coach” rehabilitation devices targeting neurological and cardiovascular diseases [44], and add further key details to support successful implementation of such a device by highlighting the importance of communicating purpose and safety, and providing ongoing technical support as essential components for user uptake and sustained use. Future work should build on the guiding principles laid out in this study, by iteratively testing and developing the prototype technology and design features with diverse users in different contexts.

**Table 2:** Guiding Principles.

User context	Key design objective or design principle	Intervention design features and implementation strategies
Acceptability: Participants expressed various preferences for the design of the technology to be acceptable to them: • Concerns around wearable sensors centred around comfort and ease of use. Particularly non-cumbersome, and easy to put on considering lack of strength, dexterity, flexibility, eyesight, and issues with proprioception. • Some participants were reluctant for their movements to be dictated by a machine. PPI group also emphasised the importance of technology to support person-centred care and not replace it. • Participants valued technology that had clear purpose. Additional considerations from the literature: • Privacy: scrutinising what data is being collected and who has access to it [45,46]. • Stigmatisation: such as prescribing exercises that are incompatible with user identity [47]. • Safety: concerns among older adults about unsupervised exercise and fear of falling [47–49]; and resilient health care approaches highlight the need to anticipate risk using a proactive systems approach which aligns with national health service patient safety standards [50,51]; human factors and ergonomics principles embedded into system design can improve patient safety [52] using tools such as human reliability analysis [53] • Financial costs of the device [49] • Trust: skepticism about device functionality [46] • Acceptability is lower where perceived added value is low [46] • Automation may help with longer term use and acceptability [46] • Behavioural enhancement has been recommended as an outcome measure rather than specific frailty measures to focus on intervention adherence in addition to clinical outcome measures since behaviour change has been associated with long term changes, and traditional frailty measures can be inconsistent [27]	• To meet the diverse physical and cognitive needs of the target user population • To clarify purpose and manage user expectations of the device	• The device must not be physically invasive or challenging to put on or wear for long periods. E.g., elasticated straps, lightweight sensors, and accessible feedback (visual and audio options) • The aims and objectives of the device need to be clear to the older adult wearer as well as the caregiver or healthcare provider supporting its use – focus should be on enhancing the therapeutic alliance rather than motivating individual behaviour change for general health and wellbeing. Key outcomes could be codesigned to maximise relevance to the different user groups and can include measures of adherence as well as clinical changes. • Through collaboration with target users with codesign methodologies, key design features should be targeted towards key motivators to use (see below), including options to select from as some exercises will be more appealing or appropriate for different people and cultures • Data management needs to be explicitly stated and consented to prior to use. • Safety of the device implementation and use within formalised healthcare practice requires a human factors and ergonomics approach to frailty management to include the device, home environment and the older adult and carer users as part of that wider system with additional safety considerations. Use of the Systems Engineering Initiative for Patient Safety (SEIPS) model recommended [54]. • Safety measures need to be built into the system for early warning of potential issues with clear instructions where adjustments or follow up by healthcare professional is needed. Safe use of the device needs to be explicitly stated to manage expectations around safety and functionality. • Where patient risk is identified, continuous improvement of safety should be enabled by assisting incident investigation through data collected and stored in the device • There should be some level of personal override to control or limit assessment and feedback, with the option to maintain fully automated settings if preferable. These settings should also include accessibility preferences for audio/visual differences.
Motivations for uptake: Participants indicated various reasons why they would be interested in a technology to monitor and encourage movements: • To improve their ability to move around independently and do the activities that are meaningful to them • To improve their sense of safety moving independently. • To improve the care they were receiving (through timely feedback, continuity of monitoring, and objective data collection). • For personalised feedback on health status (this was also important to family caregivers). • To reduce the burden on those helping them (on a personal and system level). • To oblige their healthcare professionals. • Concerns about use of the device as being overly automated and losing the sense of personhood for the user, and potentially exacerbating isolation issues, or reducing access to in-person care • Our members of the stakeholder group who were registered physiotherapists and occupational therapists saw benefit in having some objective data collection, especially for naturalistic movements because these are elements that are harder to capture in a clinical setting. • There were concerns about oversimplification and automation of physical assessments and the risk of losing the person-centred quality of their work. Additional considerations from the literature: • It would be important to manage expectations around safety, and it has been shown that healthcare providers play an important role in this regard through how they present technology and it’s intended benefits [43]	• Support safe independent movement • Collect dynamic, naturalistic biometric data that is useful to a healthcare professional • Ensure data can be summarised appropriately for user/caregiver review	• Clearly define scope and purpose of the device including inclusion and exclusion criteria for health conditions to which it could be suitable and safe • Recommended exercises are adapted to the personal needs of the user according to biometric feedback. • Allow for feedback messaging to the wearer to reassure or alert to safe/unsafe movements. • Alerts for unsafe movements trigger messaging to registered HCP or caregiver. • Option for periodic (weekly/monthly) summaries of adherence and progress (including number of concerning events) • Co-design biometric data measurement and summaries with physiotherapists, occupational therapists and primary care physicians • Co-design any safety advice accompanying the device with diverse healthcare providers including physiotherapists, occupational therapists, geriatricians, and primary care physicians using a systems approach • Provide training and support around the device functions and limitations to any clinician involved in introducing the device follow up so that the device can be used to enhance and not threaten person-based assessment and treatment
Adherence to exercise: Participants had experience of doing prescribed exercises for rehabilitation and indicated various factors that may improve their adherence: • Accountability to the therapist or facilitator • Enjoyment of exercises (especially true in a group setting) • An immediate sense of reward and/or recognition • Feeling supported and safe • Being treated respectfully as a person (non-demanding, non-patronising recommendations) Other factors to consider for longer term adherence included: • Optimal timing of exercises was personal to the participant and dependent on routine, activity levels, medication use, and participants expressed a preference that the device should have some intelligence to know when not to prompt them to do an activity • Participants felt they might want to pause adherence during temporary changes in routine such as illness, vacation, or family emergency	• To support a sense of interaction and reciprocal gain from using the device • To emphasise safety	• Incorporate principles of gamification into the design (e.g., goals and challenges, personalisation, rapid feedback, visible feedback, freedom of choice, freedom to fail, and social engagement) [55] • The device should recognise and reward activities beyond those it is programmed to deliver (e.g., active day walking around the shops should count as exercise) • The messaging of prompts needs to be co-designed to be framed well, and likely provide options for personalisation • Ensure a level of personalisation to the device (e.g., adjusting reminder frequency, allow a range of activity to be successful within minimum and maximum safety limits) • Include an ability to input data (fatigue, pain, mood) would be helpful to collect further data to inform the device to tailor more personalised feedback timing • Allow a test period where the device could pick up patterns of energy levels throughout the day (with personalisable overrides) • There should be the ability to mute the system if unable/unwilling to do the exercises at times. • The prompting system should be adaptive to the person and their context.
Technical support: Participants were willing to try a new device even if they were not generally confident in the digital domain. • Participants valued technology that were easy to use so that they felt in control of the tech rather than vice versa • Some participants noticed a decline in ability to use some devices or applications that they were previously familiar with (due to updates or cognitive changes) • Confidence and variety of tech use was often associated with having a relative or friend who could support them when needed • Confidence to use technology was impacted by frequent notifications or updates	• To keep the design as simple as possible and support users to use the device for their needs	• There needs to be accessible ongoing tech support • Simple non-digital (paper) instructions for device setup and navigation should be provided including pictures for ease of set up and use • System updates should be as infrequent as possible, with waring provided, and the option to postpone • Notifications should be limited as much as possible to safety alerts and prompts to exercise only

Given the complexity and loose definition of frailty in research and clinical practice, it is a challenging subject to address, and our data illustrates this well. Our participants were selected based on their self-reported challenges with mobility. These were revealed to be influenced by various factors including pain, loneliness, fear of falling, depression, comorbidities, and lack of strength and stability that may require different clinical approaches. Likewise, as seen in the three personas, each participant was influenced heavily by their immediate social envelope with key influences such as housing security, loneliness, financial concerns, proximity of family members, relationship to caregivers or clinicians, social engagements, faith-based community groups, cultural groups, and gender norms resulting in very different scenarios that resulted in quite different interpretations of the technology as it was described to them. In terms of behavioural intervention design, the fundamental elements of target problem (frailty) and target population (frail older adults) are problematically obscure and ill-defined forming an insecure foundation for successful intervention design [56]. These complexities suggest that for a device to be feasible, and acceptable to users, rather than designing for the needs of the older adult, which can be so varied, it should be tailored as a support tool for specific data most applicable and relevant to a healthcare professional alongside normal care, thereby adding value to the existing relationship and quality and efficiency of care provided – which ultimately was the key priority of the older adults in this study. Future iterations of this device will learn from the older adult user data generated in this study to focus on narrowing and defining the scope and purpose of the device in close collaboration with healthcare practitioners working in this area, and moving towards a more systems design approach extending routine care into the home [57]. As with the development of any continuous home health monitoring system, ongoing and careful considerations should also be made for ethical data security and privacy implications of ensuring “privacy by design” [58], including suitable consent processes [59].

This study has several limitations related to the population being studied that should be noted for future studies. Researchers noted a social desirability bias in some of the conversations that required skillful interview techniques to build rapport emphasising that the study was interested in criticism as well as agreement, and to ensure open questioning and prompting throughout. This may have been more relevant where interviews were conducted with the older adult and family caregiver at the same time, though conducting the interviews in this way was necessary for pragmatism and the comfort of the participants. Secondly, it was a challenge to describe a digital concept to older adults who may be more digitally naïve and PPI engagement was critical to assist in creating a lay standardised summary of the device design. Thirdly, participant sampling was purposive, and although some diversity was achieved in terms of ethnicity, age and frailty score, we would aim to include more cultural diversity in future studies as we noted the saliency of contextual and relationship factors in the feedback. Finally, it was noted that the frailty score used on screening (the simple frailty questionnaire [30]) did not necessarily align with the observed level of frailty upon interview (some participants appeared more frail than the frailty score suggested), and it may be relevant to adapt the screening questionnaire further to meet the needs of a particular study. It was challenging to recruit people with lower levels of frailty, and this could be an issue with self-identification and there is a potential argument to recruit via health services using clinical prefrailty indicators in the future.

## Conclusion

This paper explores older adults and their family caregivers’ perceptions of the design concept of a novel digital system designed to support prefrail-frail older adults regain strength and stability. Participants were generally enthusiastic about the idea of using the novel device alongside regular care and could see potential benefits to their own health and health service/caregiver burden. Participant feedback is presented thematically drawing out tensions between the desire to support independence while potentially threatening autonomy, and unpicking motivations to use a rehabilitation device. Diversity of context is shown in three personas generated from the data evidencing very different needs and perspectives of the device concept within the sample. A consistently noted requirement across contexts, was for technical support that needs to be easily accessible and available long-term to help older adults maintain a sense of control or self-efficacy over the device as it evolves and accounting for potential cognitive decline over time. These key influencing factors to device uptake and engagement have been included in a set of Guiding Principles with potential intervention elements to help overcome these needs including clarification of purpose, supporting safe adherence to prescribed movements, and ensuring relevant data capture and feedback to older adult users, caregivers and overseeing healthcare professionals. These guidelines can be used to help future development of this device, and others in this area, to improve the usability, acceptability, and long-term engagement necessary for the intended improved health outcomes. A systems approach to device development is recommended, shifting the focus from older adult behaviour change towards enhancing current care pathways the and patient-provider therapeutic alliance.

## Supporting information

S1 File Standardised lay summary and interview guide. (DOCX)

S2 File Summary of device design preferences. (DOCX)

S3 File COREQ checklist. (DOCX)
