# Supplementary material for: Appreciating the complexity of frailty and user context in digital health intervention design: A qualitative study with personas
Source: PLoS One. 2026 Apr 6;21(4):e0343371. doi: 10.1371/journal.pone.0343371 (PMC13052904; doi:10.1371/journal.pone.0343371)
Supplement: S1 File — (DOCX) [file pone.0343371.s001.docx]

## Supporting Information 1: Standardised lay summary and interview guide

1. Introduce study and interview:

We are a team of researchers, health professionals and technology experts. We are creating a tool to help people over 65 years old who might be finding it tricky to move around their home.

The idea is that the tool will help people move more often and more safely. This should help people keep their independence and carry on doing the things they enjoy in life.

To make sure this ends up being useful, we are trying to get a better idea about people’s experiences of moving around the home. We are also interested in what people think about this new technology. We’d like your help with that and that’s what we want to talk to you about today.

There are no right or wrong answers. This interview is just about trying to find out as much as possible in your own words.”

- **First of all, can you tell me a little bit about yourself?**
  - What do you enjoy doing?
  - Can you tell me about any movement challenges you experience?
  - What tends to help with these challenges?
  - Do you have any prescribed exercises you do at home?
  - Do you have any mobility aids that you use?

**Thank you. I’d now like to tell you a bit more about the tool we are designing**

The design is in the conceptual phases and we want to make sure it is as relevant as possible to people, like yourself, who have noticed that is getting harder to move around and it would be really helpful if we could brainstorm some of the ideas.

The concept includes using a special camera in your phone to measure your home and assess for any adaptations that may be needed to make your home safer or help you move around more easily.

- **What do you think of that initial idea?**
  - Do you have any concerns about measuring your home in this way?

To measure your movement around the home, the device includes sensors worn on the body. These could be on your wrist, like a watch, around your neck like a necklace, or worn on your waistband or in your insoles. These will detect the quality and amount of movement you are doing naturally.

- **What do you think of that initial idea?**
  - Where would you prefer the sensor to be?
  - Do you have any concerns about wearing a sensor to measure your movements?
  - Do you have any experience with health sensors?
  - How was your experience?

We would like to include a feedback system that could prompt you to move if you have been stationary for too long, and to remind you to do any exercises prescribed by your physiotherapist.

- **What do you think of that idea?**

Ideally, this device would be used to facilitate physiotherapists and occupational therapists in their treatment of people who are finding it harder to move around, and any unsafe movements or unusual physical responses can be recorded and trigger a message to these healthcare professionals to provide extra support.

- **What do you think of that idea?**

Thinking about the device now as a whole.

- **Do you have any first impressions?**
  - What do you think of the general idea of having support with moving more at home?
- **How do you feel about sharing your information with healthcare professionals via a technology like this?**
  - Can you tell me if there was anything about the description of the tool that worries you?
  - What would make you feel more comfortable?
  - Can you think of anything you’d like to change about it

**General prompt examples**

- **How does/did that make you feel?**
- **Can you tell me a little bit more about that?**
- **That’s really interesting – please can you explain a bit more?**
- **That sounds really difficult – do you feel able to tell me a bit more about t**
