# Supplementary material for: Appreciating the complexity of frailty and user context in digital health intervention design: A qualitative study with personas
Source: PLoS One. 2026 Apr 6;21(4):e0343371. doi: 10.1371/journal.pone.0343371 (PMC13052904; doi:10.1371/journal.pone.0343371)
Supplement: S2 File — (DOCX) [file pone.0343371.s002.docx]

**Supporting Information 2: Summary of device design preferences**

Legend: This is a summary of feedback from the participants directly relating to more technical features of the device for consideration at the device design stage.

**Technical Priorities**

- Comfortable: easy to put on and wear for long periods: participants were willing to wear a device as long as it is not too cumbersome.
  - Wrist: most commonly acceptable, but preferences would be for elasticated band as dexterity is an issue, and there are concerns about getting it wet and would like to be able to push it up the arm to get it out of the way.
  - Insole: several participants reported pre-existing foot issues raising concerns of putting new insoles in shoes, needing bigger shoes to fit it in, or needing to switch them between shoes
  - Waist: concerns about comfort, or weight (pulling off their centre of gravity)
- Personalisable - this applies to the style of the device and the settings e.g. frequency and style of prompts. Fatigue, mood and pain are important factors to be able to communicate to the device (sliding scale would be better than interval likert)
- Intelligent - device needs to detect when they have had an active day and provide recognition for this activity
- Simple - needs to be simple to use with limited features and ongoing accessible technology support

**Behavioural priorities**

- Motivation - overall motivation to remain independent for longer gets buy-in, but there needs to be immediate and value based motivation for engagement e.g. it is fun to use/ it will improve current care
- Reward - Many participants reported enjoying tasks that give them a reward, such as fireworks after a solitaire game, or praise
- Safety - Concerns about overdoing activities, or causing damage (especially where there is pain)

**Intervention delivery**

- Healthcare led - Participants were presented with the idea that the device would be concurrent with physio/OT services
- Caregiver monitored - Caregiving participants valued the idea of objective feedback as reassurance or early warning, as a layer between them and their GP
- The idea that there was a human monitoring the data to some degree was key
