# Supplementary material for: Appreciating the complexity of frailty and user context in digital health intervention design: A qualitative study with personas
Source: PLoS One. 2026 Apr 6;21(4):e0343371. doi: 10.1371/journal.pone.0343371 (PMC13052904; doi:10.1371/journal.pone.0343371)
Supplement: S3 File — (DOCX) [file pone.0343371.s003.docx]

**Supporting Information 3: COREQ checklist**

**Reflexivity:**

Reflexive thematic analysis explicitly recognises researcher input into the analysis. As such, it is important to declare researcher positioning. Author LD collected the data, she is a Health Psychologist and experienced qualitative researcher. Author JC helped with recruitment and screening of participants and conducted the analysis and write-up. JC is an experienced qualitative researcher with a background in health psychology and approached the data from a constructivist standpoint. Neither JC nor LD had any previous relationship with any of the participants. Supervising the study and providing input to the analysis, was KB, an experienced qualitative researcher, Health Psychologist and expert in the Person-Based Approach with over 15 years in the field of health intervention design. Analysis was iteratively revised after input from the PPIE group including those with lived and living experience of early physical frailty, caring for people with physical and cognitive frailty, including in professional capacities. The study is situated within a wider project with clinicians and an engineering team of men and women who provided technical and clinical expertise in the understanding and communication of frailty rehabilitation and the technical concept.

|  | | Item | Guide questions/description | Manuscript section where information can be found |
| --- | --- | --- | --- | --- |
| **Domain 1: Research team and reflexivity** | | | | |
| Personal Characteristics | 1 | Interviewer/facilitator | Which author/s conducted the interview or focus group? | Method- data collection |
|  | 2 | Credentials | What was their occupation at the time of the study? | Method- data collection  Supplementary material - Reflexivity |
|  | 3 | Occupation | What was their occupation at the time of the study? | Method- data collection Supplementary material - Reflexivity |
|  | 4 | Gender | Was the researcher male or female? | Supplementary material - Reflexivity |
|  | 5 | Experience and training | What experience or training did the researcher have? | Supplementary material - Reflexivity |
| Relationship with participants | 6 | Relationship established | Was a relationship established prior to study commencement? | Method – participants Supplementary material - Reflexivity |
|  | 7 | Participant knowledge of the interviewer | What did the participants know about the researcher? e*.g. personal goals, reasons for doing the research* | Method – data collection |
|  | 8 | Interviewer characteristics | What characteristics were reported about the interviewer/facilitator? e.g. *Bias, assumptions, reasons and interests in the research topic* | Supplementary material - Reflexivity |
| **Domain 2: study design** | | | | |
| Theoretical framework | 9 | Methodological orientation and Theory | What methodological orientation was stated to underpin the study? *e.g. grounded theory, discourse analysis, ethnography, phenomenology, content analysis* | Introduction Method – data analysis |
| Participant selection | 10 | Sampling | How were participants selected? *e.g. purposive, convenience, consecutive, snowball* | Method- participants |
|  | 11 | Method of approach | How were participants approached? e*.g. face-to-face, telephone, mail, email* | Method- participants |
|  | 12 | Sample size | How many participants were in the study? | Findings- participants |
|  | 13 | Non-participation | How many people refused to participate or dropped out? Reasons? | Our recruitment method does not allow us to know why participants did not respond to our invitation to participate. |
|  | 14. | Setting of data collection | Where was the data collected? e*.g. home, clinic, workplace* | Method- data collection |
|  | 15. | Presence of non-participants | Was anyone else present besides the participants and researchers? | Method- data collection |
|  | 16. | Description of sample | What are the important characteristics of the sample? *e.g. demographic data, date* | Method - participants  Table 1 |
| Data collection | 17. | Interview guide | Were questions, prompts, guides provided by the authors? Was it pilot tested? | Method- data collection |
|  | 18. | Repeat interviews | Were repeat interviews carried out? If yes, how many? | Method – data collection |
|  | 19. | Audio/visual recording | Did the research use audio or visual recording to collect the data? | Method- data collection |
|  | 20 | Field notes | Were field notes made during and/or after the interview or focus group? | Methods- data collection |
|  | 21. | Duration | What was the duration of the interviews or focus group? | Methods- data collection |
|  | 22. | Data saturation | Was data saturation discussed? | The authors are very cautious about claims of data saturation in thematic analysis (see, for example, arguments in Braun and Clarke [60]) Data saturation for the current analysis was not aimed for, instead, authors prefer the concept of information power [61] and n>20 was considered a good sample size to aim for based on our experience in previous projects. |
|  | 23. | Transcripts returned | Were transcripts returned to participants for comment and/or correction? | Member checks with participants were not conducted, professional transcribers transcribed the interviews and researchers checked for accuracy. PPIE group was a valued resource for credibility checking of analysis. |
| **Domain 3: analysis and findings** | | | | |
| Data analysis | 24. | Number of data coders | How many data coders coded the data? | Method- data analysis |
|  | 25. | Description of the coding tree | Did authors provide a description of the coding tree? | Method- data analysis.  A coding tree was not used and analysis followed reflexive thematic analysis approach and discussion with peers and PPIE. |
|  | 26. | Derivation of themes | Were themes identified in advance or derived from the data? | Method- data analysis |
|  | 27. | Software | What software, if applicable, was used to manage the data? | Method- data analysis |
|  | 28. | Participant checking | Did participants provide feedback on the findings? | Participants did not provide feedback, but ongoing analysis and reflections were discussed with the PPI group throughout the study period as stated in Method – study design section. |
| Reporting | 29. | Quotations presented | Were participant quotations presented to illustrate the themes / findings? Was each quotation identified? e*.g. participant number* | Results |
|  | 30. | Data and findings consistent | Was there consistency between the data presented and the findings? | Results |
|  | 31. | Clarity of major themes | Were major themes clearly presented in the findings? | Results  Discussion |
|  | 32. | Clarity of minor themes | Is there a description of diverse cases or discussion of minor themes? | Results |
